# The Role of Edema in Subacute Lesion Progression After Treatment of Acute Ischemic Stroke

**DOI:** 10.3389/fneur.2021.705221

**Published:** 2021-07-20

**Authors:** Praneeta Konduri, Katinka van Kranendonk, Anna Boers, Kilian Treurniet, Olvert Berkhemer, Albert J. Yoo, Wim van Zwam, Robert van Oostenbrugge, Aad van der Lugt, Diederik Dippel, Yvo Roos, Joost Bot, Charles Majoie, Henk Marquering, the MR CLEAN Trial Investigators (Multicenter Randomized Clinical Trial of Endovascular Treatment for Acute Ischemic Stroke in the Netherlands)

**Affiliations:** ^1^Department of Biomedical Engineering and Physics, Amsterdam University Medical Centers, Amsterdam, Netherlands; ^2^Department of Radiology and Nuclear Medicine, Amsterdam University Medical Centers, Amsterdam, Netherlands; ^3^Nico.lab, Amsterdam, Netherlands; ^4^Department of Radiology and Nuclear Medicine, Haaglanden Medisch Centrum, The Hague, Netherlands; ^5^Department of Neurology, Erasmus MC-University Medical Center, Rotterdam, Netherlands; ^6^Department of Radiology and Nuclear Medicine, Erasmus MC-University Medical Center, Rotterdam, Netherlands; ^7^Department of Radiology, Texas Stroke Institute, Dallas-Fort Worth, TX, United States; ^8^Department of Radiology, Cardiovascular Research Institute Maastricht, Maastricht University Medical Center, Maastricht, Netherlands; ^9^Department of Neurology, Cardiovascular Research Institute Maastricht, Maastricht University Medical Center, Maastricht, Netherlands; ^10^Department of Neurology, Amsterdam University Medical Centers, Amsterdam, Netherlands; ^11^Department of Radiology and Nuclear Medicine, Amsterdam University Medical Centers, Vrije Universiteit van Amsterdam, Amsterdam, Netherlands

**Keywords:** edema, ischemic lesion, infarct, progression, growth, post-treatment, subacute period, acute ischemic stroke

## Abstract

**Background:** Ischemic lesions commonly continue to progress even days after treatment, and this lesion growth is associated with unfavorable functional outcome in acute ischemic stroke patients. The aim of this study is to elucidate the role of edema in subacute lesion progression and its influence on unfavorable functional outcome by quantifying net water uptake.

**Methods:** We included all 187 patients from the MR CLEAN trial who had high quality follow-up non-contrast CT at 24 h and 1 week. Using a CT densitometry-based method to calculate the net water uptake, we differentiated total ischemic lesion volume (TILV) into edema volume (EV) and edema-corrected infarct volume (ecIV). We calculated these volumes at 24 h and 1 week after stroke and determined their progression in the subacute period. We assessed the effect of 24-h lesion characteristics on EV and ecIV progression. We evaluated the influence of edema and edema-corrected infarct progression on favorable functional outcome after 90 days (modified Rankin Scale: 0–2) after correcting for potential confounders. Lastly, we compared these volumes between subgroups of patients with and without successful recanalization using the Mann–Whitney *U-*test.

**Results:** Median TILV increased from 37 (IQR: 18–81) ml to 68 (IQR: 30–130) ml between 24 h and 1 week after stroke, while the net water uptake increased from 22 (IQR: 16–26)% to 27 (IQR: 22–32)%. The TILV progression of 20 (8.8–40) ml was mostly caused by ecIV with a median increase of 12 (2.4–21) ml vs. 6.5 (2.7–15) ml of EV progression. Larger TILV, EV, and ecIV volumes at 24 h were all associated with more edema and lesion progression. Edema progression was associated with unfavorable functional outcome [aOR: 0.53 (0.28–0.94) per 10 ml; *p*-value: 0.05], while edema-corrected infarct progression showed a similar, non-significant association [aOR: 0.80 (0.62–0.99); *p*-value: 0.06]. Lastly, edema progression was larger in patients without successful recanalization, whereas ecIV progression was comparable between the subgroups.

**Conclusion:** EV increases in evolving ischemic lesions in the period between 1 day and 1 week after acute ischemic stroke. This progression is larger in patients without successful recanalization and is associated with unfavorable functional outcome. However, the extent of edema cannot explain the total expansion of ischemic lesions since edema-corrected infarct progression is larger than the edema progression.

## Introduction

Treatment of acute ischemic stroke due to a large vessel occlusion aims to restore the supply of blood to the downstream ischemic tissue and cease the progression of infarction and other pathophysiological processes that result from ischemia (1, 2). Previous studies assessing ischemic and infarcted volumes on computed tomography (CT) and magnetic resonance imaging (MRI) have shown that the ischemic lesion progresses in the subacute period even after (successful) treatment, and this growth is known to be associated with unfavorable functional outcome (1–5). Previous studies have also shown that patients with unsuccessful treatment suffer from more lesion growth compared to those with successful treatment (2). Ischemic lesions as assessed on follow-up non-contrast CT (NCCT) images consist of a combination of infarct and edematous volumes. The lesion evolution may vary based on multiple factors. In patients with unsuccessful or incomplete recanalization, the evolving lesion is expected to predominantly consist of increasing infarct volume, possibly due to the expansion of infarct into the downstream territory caused by persistently reduced blood flow (2). Conversely, in patients with successful recanalization, the evolving lesions may consist of more edematous volume growth as a result of reperfusion injury and status of the microvasculature (6). Distinguishing between infarct and edema volumes (EV) may provide insight on the constituents of subacute lesion growth and help to understand the influence of subacute lesion progression on unfavorable outcome to better target secondary treatments.

Broocks et al. have developed a NCCT densitometry-based technique to quantify edema-related net water uptake within the NCCT lesion (7). In the current study, we aimed to quantify edematous and edema-corrected infarct volumes (ecIV) within the NCCT lesions at 24 h and 1 week after acute ischemic stroke using this net-water uptake based imaging biomarker. With the distinction of EV, we aimed to assess the influence of edema and edema-corrected infarct progression on favorable functional outcome after 90 days. Additionally, we also assessed the influence of successful recanalization and treatment type on edema and edema-corrected infarct progression.

## Methods

### Patient Population

In this study, we included patients that were enrolled in the Multicenter Randomized Clinical Trial of Endovascular Treatment for Acute Ischemic Stroke in the Netherlands (MR CLEAN) trial (8). Patients with an acute ischemic stroke due to a large vessel occlusion, above the age of 18 years that could be treated with endovascular treatment (EVT) within 6 h of symptom onset, were randomized to receive intravenous thrombolysis (IVT) with alteplase alone or IVT with alteplase along with EVT. More details regarding the inclusion and exclusion criterion of the trial have been provided in the study protocol (8). The MR CLEAN trial was conducted with the approval of a central medical ethics committee and the research board of each participating center. Patients or their legal representatives provided written informed consent.

**Figure 1:**
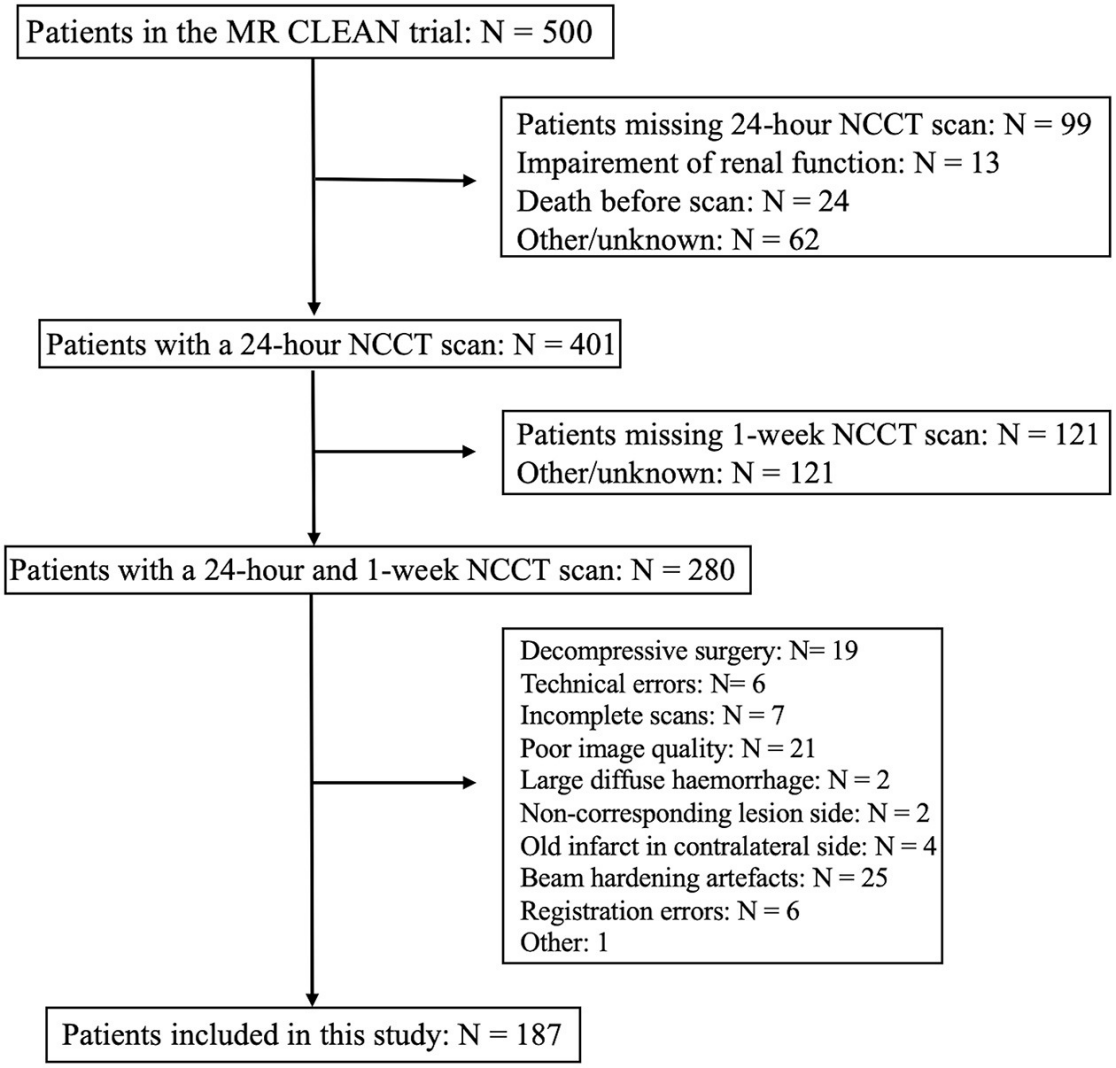
Flowchart describing the inclusion and exclusion criterion of this study.

The protocol of the MR CLEAN trial required a late follow-up NCCT scan after 1 week (variable time window of ~3–9 days) of stroke onset to evaluate the final infarct volume. Furthermore, a CT angiography scan was required 24 h after stroke onset to evaluate the post-treatment recanalization status. Performing a NCCT scan along with the CT angiography was common practice. Hence, 280 patients of the MR CLEAN trial received a NCCT scan at both time-points. In the current study, we included patients who underwent NCCT 24 h and 1 week (median: 5, IQR: 5–6 days) after onset of stroke. From these patients, we excluded those that developed a large, diffuse hemorrhages; received hemicraniectomy; or had incomplete images or images with movement artifacts, partial volume artifacts, or other technical and processing errors. From this cohort, we excluded patients with an old infarct on the contralateral hemisphere, whose images had beam hardening artifacts, registration errors, and other technical issues. Figure 1 describes the inclusion and exclusion criterion used in this study.

### Image Analysis

#### Lesion Assessment

Ischemic lesions on the 24-h NCCT scans were delineated manually on ITK Snap Software with a fixed window width of 30 Hounsfield Units (HUs) and a center-level of 35 HU by two trained observers (4). The lesion delineations included relevant hypodense areas representing edema and/or infarct expanding into the contralateral hemisphere or causing to sulcal and/or ventricular effacement. Hyperdense areas within or surrounding the lesions recognized as hemorrhages or contrast extravasation were also included. Chronic lesions with fluid attenuation, clear borders, and/or without mass effect were not included in the lesion delineation. Only the symptomatic side was revealed to the observers. Ischemic lesions on the 1-week NCCT were automatically segmented using an in-house validated software (9). One of the two experienced neuro-radiologists blinded to the clinical data, except for the symptomatic side, evaluated and corrected the lesions when required. Further details of the lesion assessment have been provided in a previous study (4). Presence of hemorrhagic transformation was assessed by the imaging core lab on the 1-week NCCT scan. The lesion delineations in the patients identified to have hemorrhagic transformations also included hyper-densities representing hemorrhagic transformation in and around the hypodense areas. The hemorrhagic areas were delineated on the 24-h (P.K) and 1-week (K.K) NCCT scans. Regions within the delineation that did not represent hemorrhagic transformation (HT) were identified as non-hemorrhagic regions.

Total ischemic lesion volume (TILV) and hemorrhagic volume (HV) were calculated as the product of number of voxels within the appropriate delineation and the voxel size.

#### Net-Water Uptake Quantification

The method developed by Broocks et al. to quantify net-water uptake involves mirroring the lesion to the contralateral hemisphere and calculating the mean density (in HU) of the ipsilateral and contralateral regions-of-interest. To automatically mirror the lesion to the contralateral hemisphere, we used an in-house NCCT atlas with centered, straight head. We first segmented the intracranial region from the 24-h and 1-week NCCT scans to exclude bone, air, and other irrelevant information (10). The segmented intracranial region and the delineated lesions were registered to the in-house atlas using Elastix software and mirrored (11–14). In patients with hemorrhagic transformation, the hemorrhagic region was excluded from the lesion. We selected voxels with a density between 20 and 80 HU from the mirrored segmentations to exclude cerebrospinal fluid and calcifications (7). The mean densities (in HU) in the ipsilateral lesion (D_ischemic_) and in the contralateral segmentation (D_normal_) were calculated. Net water uptake (NWU) per volume of lesion was the defined as (7):

$$\begin{array}{l} NWU=\;\frac{D_{normal}-\;D_{ischemic}\;}{D_{normal}} \end{array}$$

**Figure 2:**
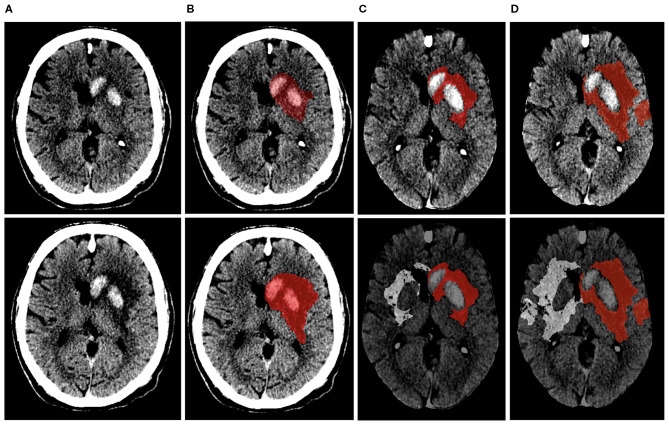
Visual representation of the steps involved in calculating net-water uptake on the 24-h (top row) and 1-week (bottom row) NCCT image of the same patient. **(A,B)** show the NCCT image and segmented lesion (represented in red). The NCCT image is registered to an in-house atlas along with the segmented lesion after excluding the hemorrhagic region **(C)**. The lesion is then mirrored to the contra-lateral hemisphere **(D)**. The NWU within the 24-h and 1-week lesion of this patient were 10% and 22%, respectively.

Figure 2 displays the steps involved in calculating net water uptake. EV was calculated as the product of the NWU and TILV (in patients without HT) and non-hemorrhagic volume (in patients with HT). ecIV was determined as the difference between TILV, EV, and HV (7). TILV, EV, HV, and ecIV progression were defined as the difference between the 1-week and 24-h TILV, EV HV, and ecIV, respectively.

### Statistical Analysis

TILV, EV, and ecIV (at 24 h and 1 week) and their progression are presented with their median and interquartile range (IQR). We compared the lesion characteristics between 24 h and 1 week using the Wilcoxon signed rank test. We performed exploratory analysis to assess the effect of 24-h lesion characteristics on EV and ecIV progression in the patient population and patient subgroups based on successful recanalization using linear regression analysis. We defined successful recanalization as modified arterial occlusion lesion (mAOL) score of three points evaluated on the 24-h CT angiography scans. Furthermore, we determined the influence of edema and edema-corrected infarct progression on using univariable and multivariable logistic regression analysis, including adjustments for potential confounders. Favorable functional outcome was assessed after 90 days and was defined as modified Rankin Scale 0–2. Baseline, clinical, and imaging and (post) treatment characteristics associated with favorable functional outcome at a significance of *p* < 0.1 were selected as potential confounders. Akaike Information Criterion was used to compare the multivariable logistic regression models with edema and edema-corrected infarct progression. Lastly, to assess the influence of successful recanalization, we compared lesion characteristics between patients that did and did not achieve successful recanalization using the Mann–Whitney *U*-test. The same test was used to assess the influence of EVT on the lesion and lesion progression characteristics.

Dichotomous and categorical baseline, clinical, and treatment characteristics of the study population are presented as proportions; normally distributed continuous variables are presented as mean ± STD and non-normally distributed continuous variables are presented as median and IQR. Patients with missing values were not included in analyses with those particular variables. Statistical analyses were performed using SPSS (IBM SPSS Statistics, version 25, 2018) and R [4.0.2 (2020-06-22) using RStudio Version 1.1.383 – © 2009-2017 RStudio, Inc.]. A *p*-value ≤ 0.05 was considered statistically significant.

## Results

### Patient Population

Of the 500 patients from the MR CLEAN trial population, we included 187 patients (Figure 1). The median age was 66 (IQR: 56–76) years, and 44% of the population was female. The median ASPECTS score was 9 (IQR: 8–10), and median NIHSS at baseline was 17 (IQR: 13–21). Ninety-one (49%) patients were randomized to receive EVT, and 100 (53%) achieved successful recanalization. Information on recanalization status was missing for 16 (8.6%) patients. Baseline characteristics are provided in Table 1.

**Table 1:** Baseline characteristics of the study population and comparison between patients with and without successful recanalization.

**Variable**		**Study population (*n* = 187)**	**Unsuccessful recanalization (*n* = 71)**	**Successful recanalization (*n* = 100)**	***p*-value**
Age (in years)		66 (56–76)	62 (51–73)	67 (57–76)	0.06
Male sex		104 (56%)	43 (61%)	55 (55%)	0.57
Previous ischemic stroke		13 (7.0%)	3 (4.2%)	10 (10%)	0.27
Myocardial infarction		23 (12%)	11 (15%)	11 (11%)	0.53
Diabetes mellitus		18 (9.6%)	6 (8.5%)	8 (8%)	1
Hypertension		94 (50%)	32 (45%)	52 (52%)	0.46
Atrial fibrillation		53 (28%)	17 (24%)	29 (29%)	0.58
Hypercholesterolemia		44 (24%)	15 (21%)	24 (24%)	0.80
Current smoking		57 (30%)	24 (34%)	31 (31%)	0.83
Antiplatelet drugs		53 (28%)	20 (28%)	28 (28%)	1
Coumarins		12 (6.4%)	1 (1.4%)	8 (8%)	0.08
Statins		54 (29%)	18 (25%)	30 (30%)	0.62
Anti-hypertensive drugs		98 (52%)	33 (46%)	54 (54%)	0.42
Systolic blood pressure (mmHg)		140 (125–160)	139 (125–160)	140 (126–158)	0.64
Clinical hemisphere side left		105 (56%)	42 (59%)	52 (52%)	0.44
Pre-stroke modified Rankin Scale (0–2)		177 (95%)	68 (96%)	94 (94%)	0.74
Baseline NIHSS		17 (13–21)	17 (13–21)	17 (13–21)	0.91
Proximal occlusion (ICA or ICA-T)		50 (27%)	20 (28%)	26 (26%)	0.89
ASPECTS score	Summary	9 (8–10)	9 (8–10)	9 (8–10)	0.51
	Missing	2 (1.1%)			
Collateral score	Absent	4 (2.2%)	1 (1.4%)	2 (2.0%)	0.19
	Filling <50% of the occluded area	51 (27%)	13 (18%)	32 (32%)	
	Filling >50% and <100% of the occluded area	69 (37%)	31 (44%)	33 (33%)	
	Filling 100% of the occluded area	62 (33%)	26 (37%)	32 (32%)	
	Missing	1 (0.54%)			
Received iv thrombolysis		169 (90%)	66 (93%)	89 (89%)	0.54
Allocated to endovascular treatment		91 (49%)	15 (21%)	69 (69%)	<0.01
Time to randomize (minutes)		190 (150–260)	200 (150–270)	190 (150–250)	0.12

*Data are displayed as median (interquartile range) or number (percentage of population). Mann–Whitney U-test and Chi-Square/Fisher-tests were performed to compare continuous and binary/categorical variables between the successful and unsuccessful recanalization sub-groups, appropriately*.

### Lesion Characteristics

In our population, the median TILV after 24 h was 37 (18–81) ml and 1 week was 68 (IQR: 30–130) ml. Median NWU was 22 (IQR: 16–26)% after 24 h and 27 (IQR: 22–32)% 1 week. Wilcoxon signed rank test showed that the TILV, EV, NWU, ecIV, and HV at 24 h were significantly lower than those at 1 week. The median TILV progression was 20 (8.8–40) ml, while the median EV progression and ecIV progression were 6.5 (2.7–15) ml and 12 (2.4–21) ml, respectively. Details of the lesion characteristics are provided in Table 2.

**Table 2:** Ischemic lesion characteristics obtained in the subacute period after treatment in 187 patients.

**Type of measurement**	**24 h**	**1 week**	**Progression**	***p*-value**
Total ischemic lesion volume (ml)	37 (18–81)	68 (30–130)	20 (8.8–40)	<0.01^**^
Net water uptake (%)	22 (16–26)	27 (22–32)	6.0 (0.33–11)	<0.01^**^
Edema volume (ml)	7.5 (2.9–17)	15 (7.9–35)	6.5 (2.7–15)	<0.01^**^
Edema corrected infarct volume (ml)	29 (15–62)	51 (21–87)	12 (2.4–21)	<0.01^**^
Hemorrhage volume (ml)	0 (0–1.3)	0 (0–2.4)	0 (0–0)	0.04^*^

*All data are displayed as median (interquartile range). Wilcoxon signed rank-test was performed between lesion characteristics obtained after 24 h and 1 week*.

** *p ≤ 0.01*.

* *p ≤ 0.05*.

### Influence of 24-h Lesion Characteristics on Lesion Progression

In our population, we found that larger EV and ecIV after 24 h were associated with larger edema and TILV progression. Similar trends were also observed in the subgroup of patients with unsuccessful recanalization. However, in the subgroup of patients with successful recanalization, the association of EV after 24 h with TILV progression was not statistically significant (*p* = 0.24). Details of the univariable linear regression are provided in Table 3.

**Table 3:** Univariable linear regression of the 24-h ischemic lesion characteristics and edema, edema-corrected infarct and lesion volume progression in the subacute period.

**Measurement**	**Population ß (95% CI) *n* = 188**	**Unsuccessful recanalizationß (95% CI)*n* = 71**	**Successful recanalization ß (95% CI) *n* = 100**
**Independent variable—Edema progression (ml)**			
Edema volume (ml)	0.37 (0.21–0.52)^**^	0.35 (0.17–0.53)^**^	0.39 (0.12–0.65)^**^
Edema corrected infarct volume (ml)	0.22 (0.17–0.27)^**^	0.23 (0.16–0.3)^**^	0.19 (0.1–0.27)^**^
Total ischemic lesion volume (ml)	0.15 (0.11–0.19)^**^	0.15 (0.1–0.2)^**^	0.13 (0.06–0.19)^**^
**Independent variable—Edema corrected infarct progression (ml)**			
Edema volume (ml)	0.05 (−0.19–0.3)	0.15 (−0.08–0.38)	0.07 (−0.46–0.60)
Edema corrected infarct volume (ml)	0.06 (−0.03–0.16)	0.1 (0–0.2)^*^	0.12 (−0.06–0.3)
Total ischemic lesion volume (ml)	0.04 (−0.03–0.11)	0.06 (−0.01–0.13)[Table-fn TN5]	0.07 (−0.06–0.2)
**Independent variable—Lesion progression (ml)**			
Edema volume(ml)	0.41 (0.05–0.78)^*^	0.49 (0.14–0.85)^**^	0.45 (−0.3–1.2)
Edema corrected infarct volume (ml)	0.28 (0.15–0.42)^**^	0.32 (0.18–0.47)^**^	0.31 (0.06–0.56)^*^
Total ischemic lesion volume (ml)	0.18 (0.09–0.28)^**^	0.21 (0.1–0.31)^**^	0.2 (0.02–0.38)^*^

** *p ≤ 0.01*.

* *p ≤ 0.05*.

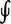
*p ≤ 0.10*.

### Influence on Favorable Functional Outcome

**Figure 3:**
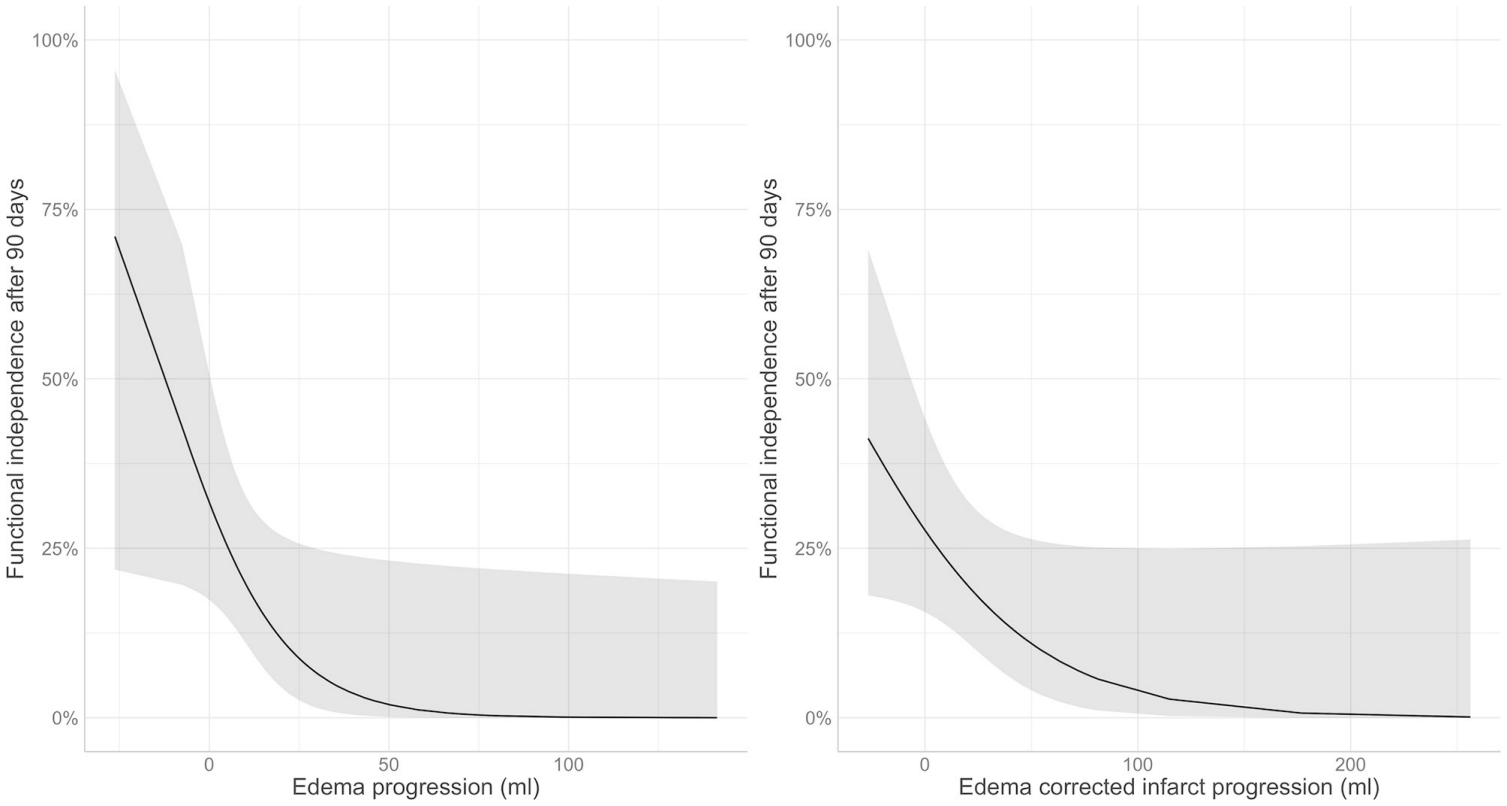
Association of post-treatment (left) edema and (right) edema corrected infarct progression in the subacute period with favorable functional outcome (modified Rankin Scale 0–2) after 90 days.

Edema and edema-corrected infarct progression were both associated with unfavorable functional outcome in the univariate analysis [OR: 0.40 (0.24–0.60); *p*-value: < 0.01; OR: 0.76 (0.62–0.91) per 10 ml; *p*-value: < 0.01]. Similar results with slightly lower significance levels were observed in the multivariable analysis including adjustments for potential confounders [aOR: 0.53 (0.28–0.94) *p*-value: 0.05; aOR: 0.80 (0.62–0.99); *p*-value: 0.06] for 10 ml of EV and ecIV progression, respectively (Figure 3). The AIC of the multivariable model with edema progression (181.5) and edema-corrected infarct progression (181.8) were comparable. Details of the multivariable logistic regression are provided in Table 4.

**Table 4:** Multivariable logistic regression analysis of edema and edema-corrected infarct progression with favorable functional outcome (modified Rankin Scale 0–2) in 187 patients.

**Variable**	**Model 1: Edema progression**		**Model 2: Edema corrected infarct progression**	
	**Odds ratio (95% CI)**	***p*-value**	**Odds ratio (95% CI)**	***p*-value**
Progression^∧^	0.53 (0.28–0.94)	0.05^*^	0.80 (0.62–0.99)	0.06[Table-fn TN9]
Total ischemic lesion volume^∧^	0.83 (0.72–0.94)	0.01^**^	0.79 (0.69–0.89)	<0.01^**^
Age	0.96 (0.93–0.99)	0.01^**^	0.96 (0.93–0.99)	0.01^**^
Coumarines	0.12 (0–1.10)	0.11	0.12 (0–1.07)	0.11
Systolic blood pressure	0.99 (0.97–1.01)	0.26	0.99 (0.97–1.01)	0.23
Baseline NIHSS	0.94 (0.87–1.00)	0.07[Table-fn TN9]	0.94 (0.88–1.01)	0.10[Table-fn TN9]
Proximal occlusion	0.42 (0.15–1.07)	0.08[Table-fn TN9]	0.38 (0.14–0.99)	0.06[Table-fn TN9]
Collaterals	1.03 (0.59–1.77)	0.92	0.94 (0.54–1.61)	0.82
Intra-arterial thrombectomy	2.29 (1.06–5.06)	0.04^*^	2.29 (1.06–5.06)	0.04^*^

∧ *Analysis done for 10 ml volume*.

** *p ≤ 0.01*.

* *p ≤ 0.05*.

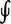
*p ≤ 0.10*.

### Influence of Treatment

**Figure 4:**
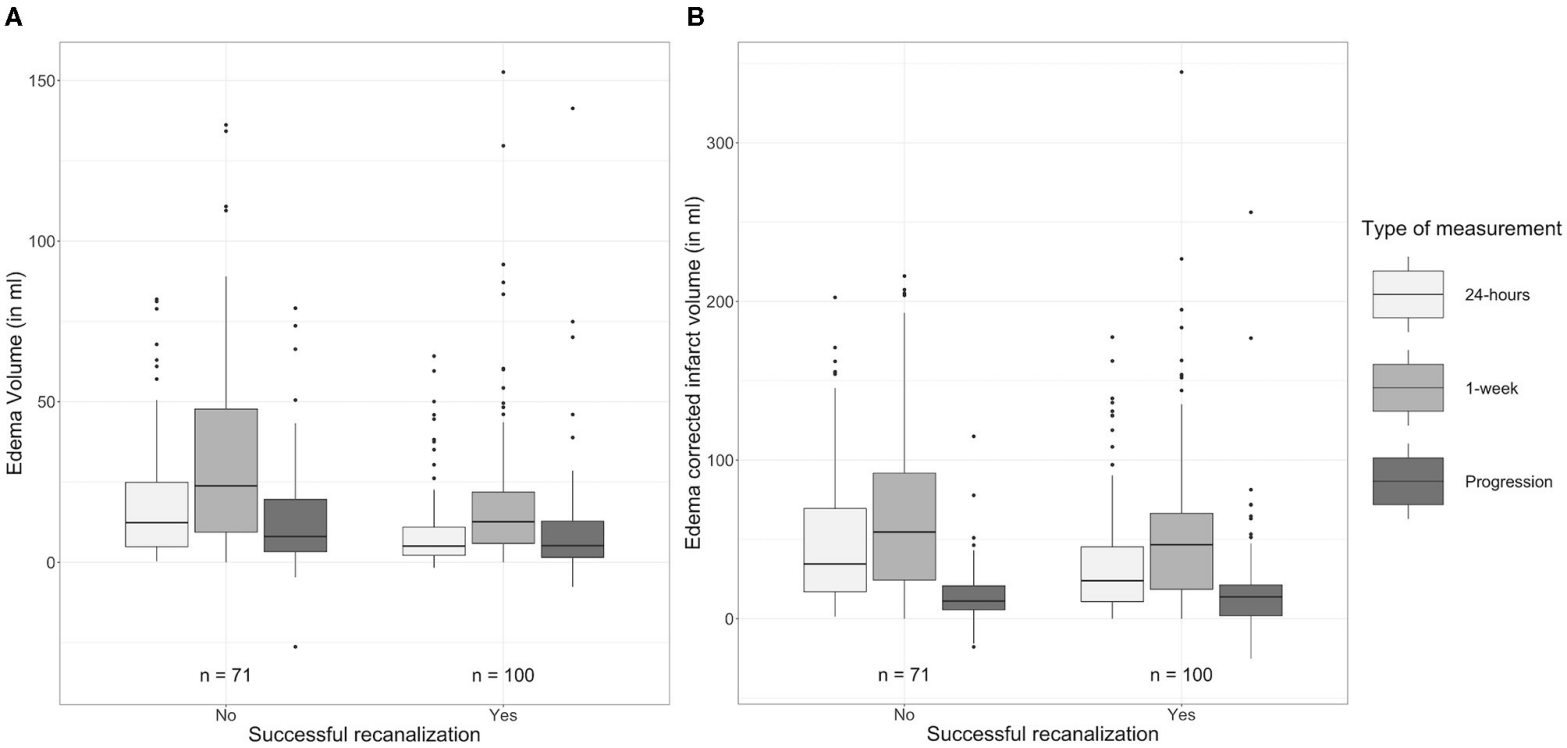
Comparison of **(A)** edema volumes and **(B)** edema-corrected infarct volumes after 24 h and 1 week after stroke onset between patients with and without successful recanalization.

As shown in Table 5 and Figure 4, patients with successful recanalization had significantly lower TILV (*p* = 0.02), EV (*p* < 0.01), and ecIV (*p* = 0.03) after 24 h compared to those without successful recanalization. Patients with successful recanalization had lower EV (*p* < 0.01) and non-significantly different ecIVs (*p* = 0.11) after 1 week. Furthermore, edema progression (*p* = 0.01) was lower in patients with successful recanalization compared to those without successful recanalization, while the ecIV progression was comparable between the subgroups (*p* = 1.00).

**Table 5:** Comparison of ischemic lesion characteristics obtained in the subacute period after treatment in patients with and without successful recanalization.

	**24 h**			**1 week**			**Progression**		
**Successful recanalization**	**No (*n* = 71)**	**Yes (*n* = 100)**	***p*-value**	**No (*n* = 71)**	**Yes (*n* = 100)**	***p*-value**	**No (*n* = 71)**	**Yes (*n* = 100)**	***p*-value**
Total ischemic lesion volume (ml)	54 (23–98)	33 (13–62)	0.02^*^	81 (35–140)	63 (24–97)	0.06	22 (10–43)	19 (5.4–33)	0.33
Edema volume (ml)	12 (4.9–25)	5.1 (2.2–11)	<0.01^**^	24 (9.4–48)	13 (5.9–22)	<0.01^**^	8.0 (3.3–20)	5.2 (1.6–13)	0.01^*^
Net water uptake (%)	24 (21–28)	20 (14–24)	<0.01^**^	30 (25–35)	25 (20–29)	<0.01^**^	5.6 (0.19–11)	6.0 (−0.04–11)	0.86
Edema corrected infarct volumes (ml)	34 (17–69)	24 (11–45)	0.03^*^	55 (24–92)	47 (19–66)	0.11	11 (5.7–21)	14 (1.9–21)	1.00
Hemorrhage volume (ml)	0 (0–1.1)	0 (0–1.3)	0.77	0 (0–2.1)	0 (0–1.8)	0.66	0 (0–0.0)	0 (0–0)	0.80

*All data are displayed as median (interquartile range). Mann–Whitney U-test was performed to assess differences in lesion characteristics between patients with and without successful recanalization (mAOL = 3 points)*.

** *p ≤ 0.01*.

* *p ≤ 0.05*.

Patients randomized to receive EVT had lower EV and percentages of NWU after 24 h (*p* = 0.01; *p* < 0.01, respectively) and lower NWU after 1 week (*p* = 0.03) compared to those who did not receive EVT. Details of the lesion characteristics compared between patients that did and did not receive EVT are provided in Table 6.

**Table 6:** Comparison of ischemic lesion characteristics obtained in the subacute period after treatment in patients that were randomized to receive EVT and iVT or only IVT.

	**24 h**			**1 week**			**Progression**		
**iVT + EVT**	**No *N*= 96**	**Yes*N* = 91**	***p*-value**	**No*N* = 96**	**Yes *N* = 91**	***p*-value**	**No *N* = 96**	**Yes*N* = 91**	***p*-value**
Total ischemic lesion volume (ml)	48 (23–81)	33 (16–77)	0.10	79 (35–130)	58 (25–99)	0.12	21 (11–43)	20 (6.8–35)	0.26
Edema volume (ml)	8.8 (5.0–20)	4.7 (2.1–13)	0.01^**^	20 (9.5–39)	14 (6.0–27)	0.07	6.7 (2.9–17)	6.1 (2.0–14)	0.36
Percentage NWU	23 (18–28)	21 (14–24)	<0.01^**^	28 (23–33)	26 (21–30)	0.03^*^	5.3 (−0.30–10)	7.0 (1.1–12)	0.15
Edema corrected infarct volume (ml)	35 (17–64)	23 (13–56)	0.14	55 (25–93)	41 (19–73)	0.20	12 (5.3–22)	12 (1.7–21)	0.32
Hemorrhage volume (ml)	0 (0–0.86)	0 (0–2.3)	0.79	0 (0–2.6)	0 (0–1.6)	0.99	0 (0–0.49)	0 (0–0)	0.07

*All data are displayed as median (interquartile range). Mann–Whitney U-test was performed to assess differences in lesion characteristics between patients with and without EVT*.

** *p ≤ 0.01*.

* *p ≤ 0.05*.

## Discussion

In this study, we showed that EV, ecIV, and hemorrhage volume continue to progress after 24 h and that larger lesions after 24 h are associated with more lesion progression in the subacute period. Furthermore, we showed that edema progression is associated with unfavorable functional outcome and that it is lower in patients who have successful recanalization.

To our knowledge, this is the first study to quantify edema progression in ischemic lesions assessed in the subacute period after stroke onset. EVs are generally described using indirect and global imaging markers like midline shift—the gold standard for assessing edema, and hemispheric, lateral ventricular, or swelling volume (15–17). Broocks et al. have developed a densitometry based NWU measure that relies on the relationship of increasing lesion volume due to water content and the associated density reduction on NCCT (18). Due to the increased reliability to distinguish EV from within the ischemic lesion compared to the global imaging markers and its easy applicability to widely available NCCT scans, this method was utilized in this study (7, 19–21).

The 24-h NWU observed in this study (22%) is comparable with the 24-h NWU presented in a previous study (20.6%) (7). We extended this finding and showed that NWU, edema, and ecIV are significantly larger after 1 week compared to those assessed 24 h after stroke onset. This indicates that the lesion progresses due to an increase in both edematous and true-infarct volumes. Ischemia and post-ischemic reperfusion deteriorate the integrity of the capillaries in the blood brain barrier, leading to more edema and ischemia. The cascading effect of the increased EV and associated rise in tissue pressure results in an increase in the mechanical forces experienced by the surrounding tissue, ultimately increasing edema and lesion volumes (22). This is supported by our finding that larger 24-h edema, edema corrected infarct, and the total lesion volume are associated with larger subacute edema and lesion progression. In this study, we calculated EV using a densitometry-based technique that measures the water content within the ischemic lesion. Hence, the EVs estimated in this study could be a marker for vasogenic edema, and not cytotoxic edema occurring due to osmotic gradients between the extra and intra-cellular spaces (22). Thus, the ecIVs are a marker for true infarct tissue, cytotoxic edema, and other ischemic components that cannot be distinguished on NCCT scans. Furthermore, we did not find that the 24-h lesion characteristics influence edema-corrected infarct progression. This may indicate that edema corrected infarct mainly extends into the downstream at-risk territory. It is also possible that edema-corrected infarct progression could be caused by other factors such as unsuccessful recanalization, no-reflow phenomenon, thrombus fragmentation, and/or formation of microvascular emboli (6). This could be supported by our finding that the 24-h lesion characteristics only showed a similar trend in the sub-population of unsuccessful recanalization. Nevertheless, the mechanism of edema-corrected infarct progression still needs to be explored.

In a previous study on this patient population, we showed that lesion progression in the subacute period is associated with unfavorable functional outcome (5). In the current study, we extended this finding to show that edema progression within the lesion is associated with unfavorable functional outcome. This finding is in line with previous studies that assessed the influence of edema on functional outcome (15, 23, 24). It is surprising that in our population, edema-corrected infarct progression only showed a similar but non-significant trend that could be due to the overlapping effect of adjusting for the 24-h lesion volume. However, due to the pertinent association between infarct and edema, a similar study on a larger population is required to validate our findings.

Successful recanalization is known to decrease edema formation. Kimberly et al. showed reduced edema, defined by midline shift, on early follow-up (24 h) and late follow-up (5–7 days) in patients with successful recanalization (15). Similarly, Broocks et al. showed that patients with successful treatment, defined as reperfusion using TICI score, have decreased NWU and associated EVs after 24 h compared to those with unsuccessful treatment (21). In addition to showing comparable results after 24 h, we showed that the similar trend continues in the subacute period up to 1 week after onset. Patients with successful recanalization demonstrated reduced EVs in comparison with patients that do not have successful recanalization in the late follow-up images as well. Furthermore, Broocks et al. also showed that growth of lesion and edema corrected infarct is lower in patients with successful treatment compared to those without successful treatment (7). Moreover, Federau et al. assessed ischemic lesions on MR perfusion images in the subacute period and showed reduced subacute lesion growth in patients that have >90% reperfusion (2). In our population, we observed similar trends in lesion and ecIVs after 24 h. We observed that total lesion and ecIV at 1 week, and lesion progression was lower in patients that have successful recanalization; however, these differences were not statistically significant. Furthermore, our finding that patients that received EVT presented with lower NWU after 24 h and 1 week further establishes the benefit of iVT and EVT over iVT only.

Our study has a few limitations. Firstly, as the 1-week NCCT scans were obtained between approximately 3 and 9 days, and since it is known that the influence of edema is most pronounced in the subacute period after stroke onset, future studies assessing the bias of the variable time frame on lesion and growth of its constituents are required (4). Secondly, mTICI score assessed on digital subtraction angiography (DSA) scans is the standard measure for reperfusion and treatment success. However, in this study, treatment success was defined as recanalization status using the mAOL score assessed on the 24-h CTA scans since DSAs were only available for the patients randomized to receive IVT and EVT. There can be a difference in the recanalization directly assessed after EVT using DSA images and assessed 24-h after treatment because of the dynamic behavior of clot formation and dissolution. Therefore, our results cannot directly be translated to current measures of treatment success. Thirdly, hyper-densities within the hypodense areas recognized as hemorrhage or contrast extravasation were included in the lesion delineations. However, hemorrhagic regions within these lesions were only defined for patients identified to be suffering from hemorrhagic transformation on the 1-week scan. Thus, the net water uptake measurements could have been biased by the influence of contrast extravasation. Lastly, 24-h lesion volume was as a potential confounder in the multivariable regression model to assess the influence of edema progression on favorable functional outcome despite being significantly associated with edema progression. This could have led to some errors associated with multicollinearity. However, since the purpose was to assess the influence of edema progression, after accounting for the TILV after 24 h, on functional outcome, both these variables were of interest and included in the final model.

## Conclusion

Both edema and ecIVs continue to progress in the subacute period after treatment of stroke, noting that lesion progression cannot be explained by increase in edema alone. Edema progression is associated with unfavorable functional outcome and is larger in patients with unsuccessful recanalization and in patients with large 24-h infarct lesions. This could also help in improving the identification of secondary treatment targets for subacute care of patients after an acute ischemic stroke.

## Data Availability Statement

The datasets presented in this article are not readily available due to ethical restrictions that prevent the sharing of patient data. Requests to access the datasets should be directed to the MR CLEAN executive committee (www.mrclean-trial.org), mrclean@erasmusmc.nl.

## Ethics Statement

The studies involving human participants were reviewed and approved by a Central Medical Ethics Committee and the research boards of all participating centers accepted the MR CLEAN trial. The patients/participants provided their written informed consent to participate in this study.

## Author Contributions

WZ, AL, RO, DD, YR, and CM designed the MR CLEAN trial. OB collected and prepared the data for the trial. PK, KK, OB, and AB prepared data for this study. PK performed the statistical analysis, interpreted the results, and drafted the paper. HM assisted with the statistical analysis, interpretation of the results, and drafting the paper. KK, AB, KT, OB, AY, WZ, RO, AL, DD, YR, JB, and CM critically revised the paper. All authors contributed to the article and approved the submitted version.

## MR CLEAN TRIAL INVESTIGATORS

Olvert A. Berkhemer, Amsterdam UMC, location AMC, Netherlands and Erasmus MC-University Medical Center Rotterdam, Netherlands. Puck S. S. Fransen, Erasmus MC-University Medical Center Rotterdam, Netherlands. Debbie Beumer, Erasmus MC-University Medical Center Rotterdam, Netherlands and Maastricht University Medical Center and Cardiovascular Research Institute Maastricht (CARIM), Netherlands. Berkhemer, Fransen, and Beumer contributed equally. Lucie A. van den Berg, Amsterdam UMC, location AMC, Netherlands. Hester F. Lingsma, Erasmus MC-University Medical Center Rotterdam, Netherlands. Albert J. Yoo, Massachusetts General Hospital, Boston, United States of America. Wouter J. Schonewille, Saint Antonius Hospital, Nieuwegein, Netherlands. Jan Albert Vos, MD, Sint Antonius Hospital, Nieuwegein, Netherlands. Paul J. Nederkoorn, Amsterdam UMC, location AMC, Netherlands. Marieke J. H. Wermer and Marianne A. A. van Walderveen, Leiden University Medical Center, Netherlands. Julie Staals, Maastricht University Medical Center and Cardiovascular Research Institute Maastricht (CARIM), Netherlands. Jeannette Hofmeijer and Jacques A. van Oostayen, Rijnstate Hospital, Arnhem, Netherlands. Geert J. Lycklama à Nijeholt and Jelis Boiten, MC Haaglanden, The Hague, Netherlands. Patrick A. Brouwer and Bart J. Emmer, Erasmus MC-University Medical Center Rotterdam, Netherlands. Sebastiaan F. de Bruijn and Lukas C. van Dijk, HAGA Hospital, The Hague, Netherlands. L. Jaap Kappelle, University Medical Center Utrecht, Netherlands. Rob H. Lo, University Medical Center Utrecht, Netherlands. Ewoud J. van Dijk and Joost de Vries, Radboud University Medical Center, Nijmegen, Netherlands. Paul L. M. de Kort and Willem Jan J. van Rooij, Sint Elisabeth Hospital, Tilburg, Netherlands. Jan S. P. van den Berg and Boudewijn A. A. M. van Hasselt, Isala Klinieken, Zwolle, Netherlands. Leo A. M. Aerden and René J. Dallinga, Reinier de Graaf Gasthuis, Delft, Netherlands. Marieke C. Visser and Joseph C. J. Bot, Amsterdam UMC, location VU, Amsterdam, Netherlands. Patrick C. Vroomen and Omid Eshghi, University Medical Center Groningen, the Netherlands. Tobien H. C. M. L. Schreuder and Roel J. J. Heijboer, Atrium Medical Center, Heerlen, Netherlands. Koos Keizer and Alexander V. Tielbeek, Catharina Hospital, Eindhoven, Netherlands. Heleen M. den Hertog and Dick G. Gerrits, Medical Spectrum Twente, Enschede, Netherlands. Renske M. van den Berg-Vos and Giorgos B. Karas, Sint Lucas Andreas Hospital, Amsterdam, Netherlands. Ewout W. Steyerberg, Erasmus MC-University Medical Center Rotterdam, the Netherlands. H. Zwenneke Flach, Isala Klinieken, Zwolle, Netherlands. Henk A. Marquering and Marieke E. S. Sprengers, Amsterdam UMC, location AMC, Netherlands. Sjoerd F. M. Jenniskens, Radboud University Medical Center, Nijmegen, Netherlands. Ludo F. M. Beenen and René van den Berg, Amsterdam UMC, location AMC, Netherlands. Peter J. Koudstaal and Wim H. van Zwam, Erasmus MC-University Medical Center Rotterdam, Netherlands. Yvo B. W. E. M. Roos, Amsterdam UMC, location AMC, Netherlands. Aad van der Lugt, Erasmus MC-University Medical Center Rotterdam, Netherlands. Robert J. van Oostenbrugge, Maastricht University Medical Center and Cardiovascular Research Institute Maastricht (CARIM), the Netherlands. Charles B. L. M. Majoie, Amsterdam UMC, location AMC, Netherlands. Diederik W. J. Dippel, Erasmus MC-University Medical Center Rotterdam, Netherlands.

## Conflict of Interest

PK was funded by INSIST (www.insist-h2020.eu): a European Union's Horizon 2020 research and innovation programme (Grant Agreement Number: 777072). AB is a shareholder of Nico.Lab. AY reports grants from Cerenovus Neurovascular, Medtronic, Stryker, Penumbra, and Genentech for investigator-initiated studies; funds from Stryker, Cerenovus Neurovascular and Penumbra (core imaging lab activities) and Genentech (consultation); and declares to have equity ownership from Insera Therapeutics. WZ reports speaker fees from Stryker and Cerenovus (paid to the institution). AL and DD report funds from the Cerenovus Neurovascular, Dutch Heart Foundation, Brain Foundation Netherlands, Organization for Health Research and Development, Health Holland Top Sector Life Sciences and Health, and unrestricted grants paid to the institution from AngioCare BV, Covidien/EV3, MEDAC Gmbh/LAMEPRO, PenumbraInc., Top Medical/Concentric, Stryker, Stryker European Operations BV, Medtronic, Thrombolytic Science and LLC for research. AL further reports grants paid to the institution from the Siemens Healthineers, GE Healthcare, and Philips Healthcare. YR is a shareholder at Nico.Lab. CM reports grants from European Commission, during the conduct of the study; grants from CVON/Dutch Heart Foundation, grants from TWIN Foundation, grants from Stryker, outside the submitted work; and owns stock in Nico.lab. HM is a co-founder and shareholder of Nico.lab. The remaining authors declare that the research was conducted in the absence of any commercial or financial relationships that could be construed as a potential conflict of interest.

## Abbreviations

CT: Computed Tomography
MRI: Magnetic Resonance Imaging
NCCT: Non-Contrast Computed Tomography
MR CLEAN: Multicenter Randomized Clinical Trial of Endovascular Treatment for Acute Ischemic Stroke in the Netherlands
EVT: Endovascular Treatment
IVT: Intravenous Thrombolysis
HU: Hounsfield Unit
HT: Hemorrhagic Transformation
TILV: Total Ischemic Lesion Volume
HV: Hemorrhagic Volume
NWU: Net water uptake
EV: Edema Volume
ecIV: Edema Corrected Infarct Volume
IQR: Inter Quartile Range
mAOL: modified Arterial Occlusion Lesion
NIHSS: NIH Stroke Scale/Score
ASPECTS: Alberta Stroke Program Early Computed Tomography Score
ICA: Intracranial carotid artery
ICA-T: Intracranial carotid artery-T junction
TICI: Thrombolysis in Cerebral Infarction.
